# Tulane virus recognizes sialic acids as cellular receptors

**DOI:** 10.1038/srep11784

**Published:** 2015-07-06

**Authors:** Ming Tan, Chao Wei, Pengwei Huang, Qiang Fan, Christina Quigley, Ming Xia, Hao Fang, Xufu Zhang, Weiming Zhong, John S. Klassen, Xi Jiang

**Affiliations:** 1 Division of Infectious Diseases, Cincinnati Children’s Hospital Medical Center, Cincinnati, OH; 2 Department of Pediatrics, University of Cincinnati College of Medicine, Cincinnati, OH; 3 School of Traditional Chinese Medicine, Southern Medical University, Guangzhou, China; 4 Alberta Glycomics Centre and Department of Chemistry, University of Alberta, Edmonton, Alberta, Canada

## Abstract

The recent discovery that human noroviruses (huNoVs) recognize sialic acids (SAs) in addition to histo-blood group antigens (HBGAs) pointed to a new direction in studying virus-host interactions during calicivirus infection. HuNoVs remain difficult to study due to the lack of an effective cell culture model. In this study, we demonstrated that Tulane virus (TV), a cultivable primate calicivirus, also recognizes SAs in addition to the previously known TV-HBGA interactions. Evidence supporting this discovery includes that TV virions bound synthetic sialoglycoconjugates (SGCs) and that treatment of TV permissive LLC-MK2 cells with either neuraminidases or SA-binding lectins inhibited TV infectivity. In addition, we found that *Maackia amurensis* leukoagglutinin (MAL), a lectin that recognizes the α-2,3 linked SAs, bound LLC-MK2 cells, as well as TV, by which MAL promoted TV infectivity in cell culture. Our findings further highlight TV as a valuable surrogate for huNoVs, particularly in studying virus-host interactions that may involve two host carbohydrate receptors or co-receptors for infection.

Caliciviruses are a group of nonenveloped RNA viruses containing a single-stranded, positive-sense RNA genome in the family *Caliciviridae*. These viruses are divided into six major genera with diverse host tropisms causing variable diseases in both humans and animals. Human noroviruses (huNoVs), major members of the *Norovirus* genus in the Calicivirus family, are the most important viral pathogens of epidemic acute gastroenteritis affecting people of all age groups in both developed and developing countries^1^. The US CDC estimates that huNoVs are responsible for up to 21 million illnesses, 1.9 million outpatient visits, 400,000 emergency department visits, 71,000 hospitalizations, and 800 deaths each year in the United States^2^, a fraction of the 218,000 deaths worldwide annually^3^. In spite of the significant impact on public health, our understanding of huNoVs remains limited, owing to the absence of a robust cell culture system and an effective small animal model, despite the recent progress in culturing huNoVs in BJAB cells^4^ and the development of an immunocompromised mouse infection model^5^ for huNoVs. As a result, there are no specific prophylactic or therapeutic approaches available against huNoV-associated diseases to date. Current investigation into huNoV-host interactions relies mainly on *in vitro* binding experiments using recombinant huNoV capsid proteins, including virus-like particles (VLPs)^6^ and P particles^7^,^8^, and cultivable caliciviruses as huNoV surrogates.

Tulane virus (TV), the prototype of the *Recovirus* genus in the Calicivirus family, was first identified in stool samples from rhesus macaques^9^,^10^. TV shares several important features with huNoVs, including the same genetic organization^9^ and similar capsid structure^11^. Most importantly, both huNoVs and TV are enteric viruses leading to gastroenteritis^12^ and recognize histo-blood group antigens (HBGAs) as attachment factors for infection^10^,^13^. Thus, TV is considered an excellent surrogate for studying huNoV-host interactions, particularly for understanding the process of viral attachment and entry into host cells through interaction with cellular receptors and/or co-receptors.

Attachment to a host cell receptor is the first, essential step for a virus to initiate a successful infection. The surfaces of the intestinal epithelial cells are covered by a thick layer of glycocalyx consisting of various glycans and many viral pathogens are known to recognize specific carbohydrates for attachment and entry. For example, huNoVs recognize HBGAs for attachment in a strain-specific manner^14^,^15^,^16^ and a number of huNoV-HBGA binding profiles have been described^6^,^17^. The structural bases of huNoV-HBGA interactions have been elucidated through crystallography studies^18^,^19^,^20^,^21^, while site-directed mutagenesis experiments confirmed the HBGA-binding sites based on mutated VLP and P particle lost binding to HBGAs^18^,^20^,^21^,^22^,^23^,^24^. Furthermore, the roles of huNoV-HBGA interactions in host susceptibility have been shown through human volunteer challenge studies^25^,^26^,^27^ and huNoV outbreak investigations^28^,^29^,^30^,^31^,^32^,^33^,^34^. However, under *in vitro* conditions huNoVs cannot replicate in HBGA-expressing cell lines derived from human intestine, indicating another cell factor that is required for huNoV infection is missing. In fact, the interactions between huNoVs and non-HBGA factors have been reported. For example, earlier studies showed that huNoV VLPs interacted with heparan sulfates on the cell surface^35^ and an undefined 105-kDa membrane protein^36^. In a separate study, huNoVs were found to recognize sialyl Lewis x, sialyl diLewis x, and sialylated type 2 chain conjugates^37^. Furthermore, a recent study indicated that huNoVs recognize gangliosides as ligands, in which the sialic acid (SA) components played an important role^38^. These data strongly suggest that non-HBGA factors and SA-containing sialoglycoconjugates (SGCs) may be other important host factors for huNoV infection. In this study, we provide further evidence supporting the importance of SGCs in calicivirus infection by studying an animal calicivirus, the Tulane virus, as a surrogate for huNoVs.

SGCs are major components of the surface glycocalyx of intestinal epithelial cells. SAs are typically present as outermost monosaccharides of SGCs, including glycolipids, glycoproteins, glycophospholipid anchors, and proteoglycans, which have important roles in diverse physiological and pathological processes^39^. Numerous viral pathogens are known to recognize SAs of SGCs as attachment factors or receptors for infection, including influenza virus^40^,^41^, paramyxovirus^42^, reovirus^43^,^44^, and picornavirus^45^. Within *Caliciviridae*, feline calicivirus (FCV) from the *Vesivirus* genus^46^, porcine sapovirus (PSaV) from the *Sapovirus* genus^47^, and murine norovirus (MNV) from the *Norovirus* genus^48^ have also been shown to recognize SAs as receptors for infection, suggesting that SGCs may be commonly recognized by caliciviruses.

In addition to evidence for TV recognizing SGCs by *in vitro* binding experiments, we have demonstrated in this study that the infection of TV in cell cultures is significantly reduced by treatment of the host cells with either neuraminidases or SA-binding lectins. We also found that *Maackia amurensis* leukoagglutinin (MAL), a commercially available lectin that recognizes α-2,3 linked SAs, increased TV infectivity in cell culture. Since huNoV-SGC interactions have also been reported^37^,^38^, our new data support the notion that the cultivable TV is an excellent huNoV surrogate to study huNoV-host interactions.

## Materials and Methods

### TV and LLC-MK2 cells

TV was originally isolated from rhesus macaque stool samples^9^; lab stock was used in the current study. The TV-permissive cell line, LLC-MK2, is a rhesus macaque (Macaca mulatta) kidney-derived cell line that was originally purchased from ATCC (Manassas, VA). The cells were cultured in 199 medium (Mediatech, Manassas, VA) supplemented with 10% fetal bovine serum (FBS), 100 U/mL penicillin G, and 100 μg/mL streptomycin (Life Technologies, Grand Island, NY), at 37 °C, 5% CO2, and 100% relative humidity.

### TV purification

TV was purified and quantified as described previously^13^. Briefly, LLC-MK2 cells were cultivated in rolling bottles. At about 85% confluence, cells were inoculated with 10 ml of TV inoculum (MOI = 0.4). After 60 minutes, the inoculum was replaced by 50 ml of medium without FBS. After further culture for 72 hours, the culture media were harvested by centrifugation (5,000 × *g*, 10 minutes) to remove cells and cell debris. The supernatants were pooled (~600 ml). TVs were pelleted down by centrifugation at 141,118 × *g* for 2 hours. TV pellets were resuspended in 3 to 5 mL 1x phosphate buffer saline (PBS, pH7.4). After adjustment of the TV suspension to a density of 1.365 (refractive index) with cesium chloride (CsCl), centrifugation was performed at 288,000 × *g* for 40 hours. The CsCl gradient was fractionated into 22 fractions through bottom puncture of the centrifugation tubes. TV containing fractions were monitored and quantified by electron microscopy (EM), SDS-PAGE, western-blot, reverse transcription-PCR (RT-PCR), and plaque assays (see below). Factions corresponding to the purified TV from a CsCl gradient containing cells/media without TV infection were also collected (designated as cell lysate) for control purpose.

### TV-SGC binding assays

ELISA-based binding assays were performed using various synthetic, biotinylated SGCs. Purified TVs at a 1:40 dilution (containing TVs at ~1 × 10^4 ^PFU/mL), cell lysate without TV (see above) at a 1:40 dilution, recombinant HA protein (10 μg/mL) of an H3N1 influenza virus were coated on microtiter plates overnight at 4 °C. After blocking with nonfat milk, the plates were incubated with various synthetic, biotinylated SGCs/HBGAs (1 μg/mL) and the bound SGCs/HBGA were detected by streptavidin-HRP conjugates (Jackson Immuno Research, West Grove, PA). The signal intensity was measured using the optical density at a wavelength of 450 nm (OD_450_). Synthetic oligosaccharides *N*-acetylneuraminic acid (Neu5Ac or NANA), N-Glycolylneuraminic acid (Neu5Gc), 3’-sialyllactose (Neu5Ac-α2,3-Gal-β1,4-Glc), 6’-sialyllactose (Neu5Ac-α2,6-Gal-β1,4-Glc), 6’-sialyllacNAc (Neu5Ac-α2,6-Gal-β1,4-GlcNAc), B-trisaccharide [Gal-α1-3-(Fuc-α1-2)-Gal], and A disaccharide (GalNAc-α1-3-Gal) were purchased from GlycoTech (Rockville, MD). All of these oligosaccharides were conjugated with polyacrylamide (PAA) and were biotinylated. Each experiment was repeated at least four times.

### TV plaque assay

This was performed on LLC-MK2 cell monolayers in 12-well plates. Cells were seeded in the wells and incubated at 37 °C with 5% CO_2_ one day prior infection. For TV infection, 0.5 mL diluted virus stock (at desired titers) was added to each well and incubated for 60 minutes on a rotating platform at 37 °C. The media were then removed and cell monolayers were washed once with PBS, and then overlaid with 1.0 mL culture media containing 0.75% agarose. The plates were further incubated at 37 °C for 3 days. The cells were then fixed with 15% formaldehyde in PBS for 15 minutes, and the cell monolayer was stained with 0.02% Crystal Violet solution (Sigma, St. Louis, MO). For blocking experiments, either TV inoculations or seeded cells were pretreated with various blocking reagents, including neuraminidases and SA binding lectins (see below), either separately or in combination, as indicated.

### TV plaque assays with Lectin, and/or neuraminidase treatments

For treatment of LLC-MK2 cells with lectins, *Maackia amurensis* leukoagglutinin (MAL) (Sigma, St. Louis, MO) and *Sambucus nigra* lectin (SNL), (Sigma, St. Louis, MO) were diluted to 100 μg/mL in 1 × PBS as stock solution. Prior to TV infection, culture media were replaced by 500 μL of diluted lectin at indicated concentrations and incubated on a rotary shaker at 37 °C for 60 minutes in a CO_2_ incubator. The mock controls were treated with 1 × PBS only. After incubation, 500 μL of TV inoculation at desired titers were added for plaque assays (see above). The experiments were repeated for six times (N = 6). Treatment of LLC-MK2 cells with SNL or MAL at the studied concentrations did not affect the viability of the cells as monitored by microscopy.

For treatment of LLC-MK2 cells with either neuraminidase of *Vibrio cholera* (VCNA) or *Arthrobacter ureafaciens* (AUNA) (Roche, Indianapolis, IN), the enzyme was first diluted in 1 × PBS (pH5.5) with 1mM CaCl_2_, or with the dilution buffer provided by the manufacturer. Both enzymes hydrolyze terminal N- or O-acyl-neuraminic acids in α-2,3, α-2,6, or α-2,8 linkage, but preferentially cleaves α-2,3 and α-2,6 linkages, respectively, at higher rate than other linkages. Prior to TV infection, culture media were replaced by 500 μL of neuraminidase solution at 60 mU/well. The cells were further incubated as described above on a shaker in a CO_2_ incubator. The mock controls were treated with the dilution buffer only. The neuraminidase solutions were then removed and cells were washed once with PBS. TV inoculations at desired titers were added for plaque assays. The experiments were repeated for six times (N = 6). Treatment of LLC-MK2 cells with either VCNA or AUNA at the studied concentrations and conditions did not affect the viability of the cells as monitored by microscopy.

### TV-lectin binding assays

Purified TV diluted 1:40 (containing TVs at ~1 × 10^4^ PFU/mL) and the corresponding fraction of cell lysate without TV at the same dilution was coated on microtiter plates overnight at 4 °C (see above). The coated TVs were blocked for 60 minutes with human milk supernatant after centrifugation of 5,000–10,000 rpm for 5 minutes. Cow milk and nonfat milk are not suitable blocking reagents for SNL lectin, as SNL recognizes unknown component(s) of cow milk. Biotinylated MAL or SNL lectin at 10 μg/mL was incubated with coated TV for 60 minutes or longer time of overnight. The bound MAL or SNL lectin was detected by streptavidin-HRP conjugates (Jackson ImmunoResearch, West Grove, PA). The signal intensity was measured at OD_450_. The same fractions from a CsCl gradient of uninfected LLC-MK2 cells corresponding to the tested TV-containing fractions were used as negative controls.

**Growth curves of TV with and without MAL treatment.** The protocol for a single-cycle growth curve of TV was modified from a previous publication^49^. Briefly, LLC-MK2 cells in 6-well tissue culture plates (1 × 10^6^ cells/well) were incubated with MAL (100 ug/mL/well in 1 × PBS) or 1 × PBS only at 37 °C in a CO_2_ incubator on a rotary shaker for 60 minutes. 1 mL TV stock in 2x culture media (MOI = 1) was added to each well, and the cells were cultured further at 37 °C in a CO_2_ incubator. At each time point (0, 4, 8, 12, 20, and 28 hours post-infection), culture media were replaced by 0.5 mL Trizol solution (Life Technologies), the cells on plates were stored at 4°C until all samples were collected. Total RNA was extracted from each well for quantitative RT-PCR (qRT-PCR) analysis (see below). The experiments were done in triplicate.

**qRT-PCR analysis.** The total TV RNAs from cell samples above with or without MAL-treatment were extracted with Trizol solution and qRT-PCR was performed to determine the effects of MAL on TV replication using the TaqMan® One-Step RT-PCR Master Mix Reagents Kit. Briefly, 100 ng of total RNA was used for reverse transcription and amplification with the Applied Biosystems® 7500 Fast Real-Time PCR System (Life Technologies). The specific primer pair (TCGCGCAGCGCACTTA and CAAGAATCCAGAACAACCAATATCA) for amplification of a fragment of TV RNA-dependent RNA polymerase (RdRp) gene was synthesized by Integrated DNA Technologies, Inc (IDT, Coralville, Iowa) and the probe targeting same region of TV was purchased from Life Technologies. qRT-PCR reactions using RNAs extracted from TVs collected in each time point were performed in duplicate in following program: 50 °C × 1, 5 minutes; (95 °C × 1, 20 seconds; 95 °C, 30 seconds and 60 °C, 30 seconds) × 40 cycles. The Applied Biosystems® Eukaryotic 18S rRNA (4319413E, Life Technologies) was used as an endogenous control for calculating the fold change by 2^∆∆Ct.

### Fluorescence microscopy

The LLC-MK2 cells were seeded on coverslips one day before the experiment. For detection of α-2,3 linked SA distribution on cell surface, fluorescein-labeled MAL (1:500 dilution in DMEM) was incubated with cells for 60 minutes at 37 °C. Unbound MAL was removed by washing the cells three times with 1 × PBS (pH7.4). The fluorescent signals from stained cells were observed on an inverted fluorescent microscope (ApoTome fluorescence microscope, Zeiss) and representative pictures were taken. To monitor the dynamic movements of the bound MAL on LLC-MK2 or MA-104 (another primate kidney cell line that supports TV replication) cells, fluorescein-labeled MAL was incubated with cells for different lengths of time (15, 30, 45, 60,120, and 240 minutes) before fluorescence microscopy. For detection of TV replication in LLC-MK2 cells, cells were fixed with 4% paraformaldehyde and permeabilized by 0.2% Triton X-100 about 20 hours post-infection. Then, TVs were stained with our in-house anti-TV rabbit hyperimmune serum. Rhodamin-conjugated donkey anti-rabbit IgG (Jackson ImmunoResearch Inc.) was used to visualize TV signals. Cell nuclei were stained with 4′,6-Diamidino-2-phenylindole dihydrochloride (DAPI) (Vector Laboratory Inc.).

### Detection of HBGAs on cells

LLC-MK2 cells were grown to confluence on coverslips and fixed with 1% paraformaldehyde for 15 min. The fixed preparations were blocked with 3% BSA for 60 min and then incubated separately with a panel of monoclonal antibodies (Mabs) specific to A, B, H1, H2, Le^a^, Le^b^, Le^x^, and Le^y^ (Covance Inc., except the Mab against B antigen from Accurate Chemical and Scientific Corporation), at a final dilution of 1:100 at 37 °C for 60 min. FITC-conjugated goat anti-mouse IgG (Calbiochem Inc.) at a 1:400 dilution was applied for 60 minutes. Finally, the coverslips were stained with DAPI and mounted with Vectashield (Vector Laboratories). The stained cells were observed on an inverted fluorescent microscope, as described above.

### Graph preparation and statistical analysis

Graphs were prepared using GraphPad Prism 6 (GraphPad Software, Inc.) and Excel 2010 (Microsoft Corporation). Statistical differences among data groups were analyzed in GraphPad Prism 6 using unpaired t-test. *P* values were set at 0.05 (P < 0.05) for significant difference (*), 0.01 (P < 0.01) for highly significant difference (**), and 0.001/0.0001 (P < 0.001/P < 0.0001) for extremely significant difference (***/****).

## Results

### TV interacts with SGCs *in vitro*

**Figure 1:**
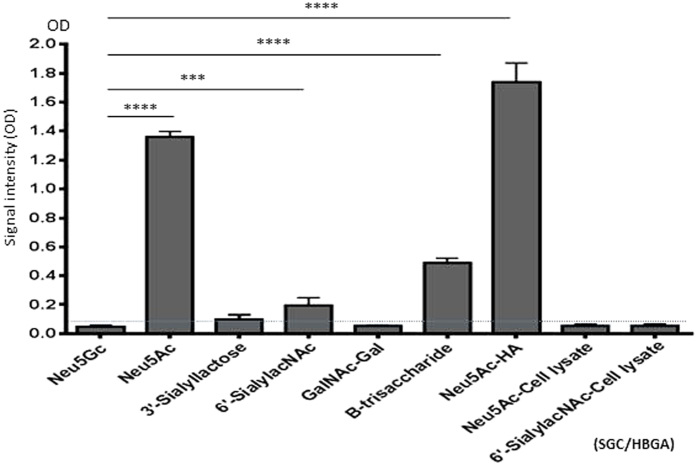
Purified Tulane virus (TV) virions interact with some synthetic sialoglycoconjugates (SGCs) in ELISA-based binding assays. Cesium chloride gradient-purified TV virions (1 × 10^4^ PFU/mL) were coated on microtiter plates and incubated with various synthetic SGCs or oligosaccharides (1 μg/mL, X-axis), including *N*-acetylneuraminic acid (Neu5Ac), *N*-glycolylneuraminic acid (Neu5Gc), 3’-sialylactose, 6’-sialyllacNAc, type A disaccharide (GalNAc-α1-3-Gal), and type B trisaccharide. All of these SGCs and oligosaccharides are linked to polyacrylamide (PAA) and biotinylated. The bound SGCs and oligosaccharides were detected by streptavidin-horseradish peroxidase (HRP) conjugates. Binding signals were shown as optical density (OD_450_, Y-axis). Neu5Gc, 3’-sialylactose, and type A disaccharide were negative controls, while type B trisaccharide and the interaction between Neu5Ac and the recombinant HA protein (10 μg/mL, coated on plate) of an H3N1 influenza virus (Neu5Ac-HA) were positive controls. The binding of the cell lysate without TV with Neu5Ac (Neu5Ac-cell lysate) or 6’-sialyllacNAc (6’-sialyllacNAc-cell lysate) are also negative controls. A dashed line indicates the signal cutoff of 0.1. Each binding experiment was repeated at least four times. The statistical differences between the binding results and the negative control (Neu5Gc) are indicated.

Both huNoVs and TV are known to interact with HBGAs^6^,^13^. Following our recent discovery that huNoVs also recognize SGCs^38^, we performed ELISA binding assays using purified TV virions and different synthetic SGCs. The results (Fig. 1) showed that TV bound strongly to the *N*-Acetylneuraminic acid (Neu5Ac or NANA) (P < 0.0001), and weakly to 6’-sialyllacNAc with a α-2,6 linked SA (P = 0.0011), but not to *N*-Glycolylneuraminic acid (Neu5Gc), 3’-sialylactose with a α-2,3 linked SA and a type A disaccharide GalNAc-Gal without a SA that served as negative controls. Two further controls were performed using the gradient faction of uninfected LLC-MK2 cell lysate corresponding to the purified TV fraction to bind Neu5Ac (cell ysis-Neu5Ac) and 6’-sialyllacNAc (cell ysis-6’-sialyllacNAc) (Fig. 1). The negative results excluded the possibility of the observed binding signals from contaminated cellular proteins. A common feature of the binding SGCs is their SA residues, although the types, locations, and linkages of the SAs clearly affect the bindings. As positive controls, TV virions bound type B trisaccharide and recombinant HA protein of an H3N1 influenza virus bound strongly Neu5Ac (Neu5Ac-HA).

### Neuraminidase treatment of host cells inhibited TV infectivity

**Figure 2:**
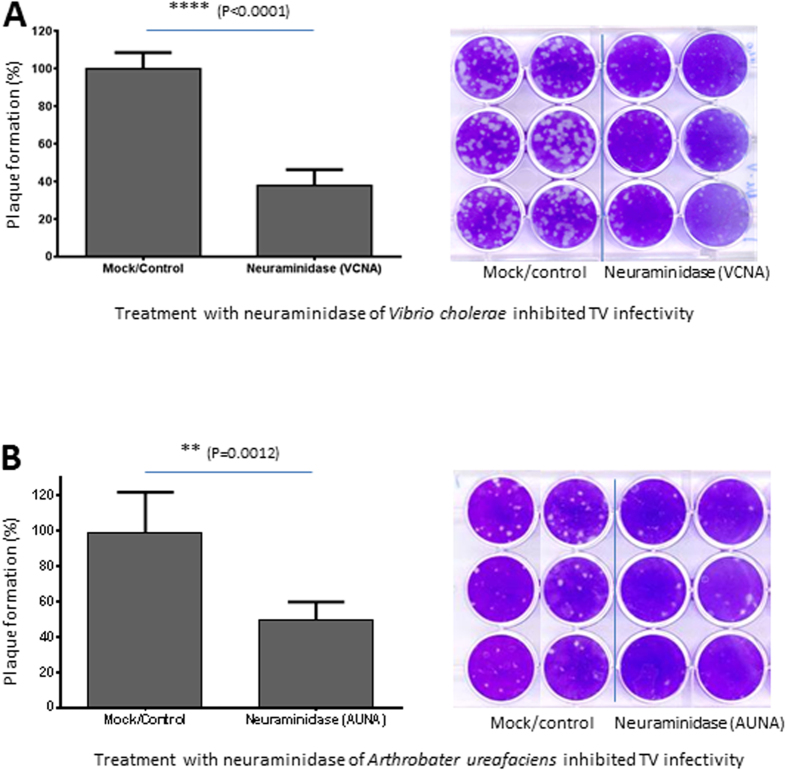
Neuraminidase treatment reduces TV infectivity in LLC MK2 cells. Cells were treated with neuraminidases (60 mU/well) from *Vibrio cholerae* (VCNA) (**A**) or *Arthrobacter ureafaciens* (AUNA) (**B**) prior to TV infection. Mock control cells were treated with PBS only. The mean values (plaque numbers) of the mock/control group were set as 100%, while percentages (%) of the plaque numbers for individual wells of both mock/control and neuraminidase-treatment groups relative to the mean values of the mock/control group were calculated and shown. Each experiment was repeated at least six times. The statistical differences between data groups are indicated. Representative plaques/wells of both mock/control and neuraminidase-treatment groups are shown.

To further determine the biological significance of SAs in TV infection, TV-permissive LLC-MK2 cells were treated separately with two neuraminidases from *Vibrio cholerae* (VCNA) and *Arthrobater ureafaciens* (AUNA) prior to TV infection for plaque assays. Treatment with neuraminidases at 60 mU/well significantly reduced TV infection by 63.3% with VCNA (Fig. 2A, *P* < 0.0001) and by 50.4% with AUNA (Fig. 2B, *P* = 0.0012), compared to untreated cells. These results further support the importance of cell surface SAs in TV infection.

### Effects of SA-binding lectins on TV infectivity

**Figure 3:**
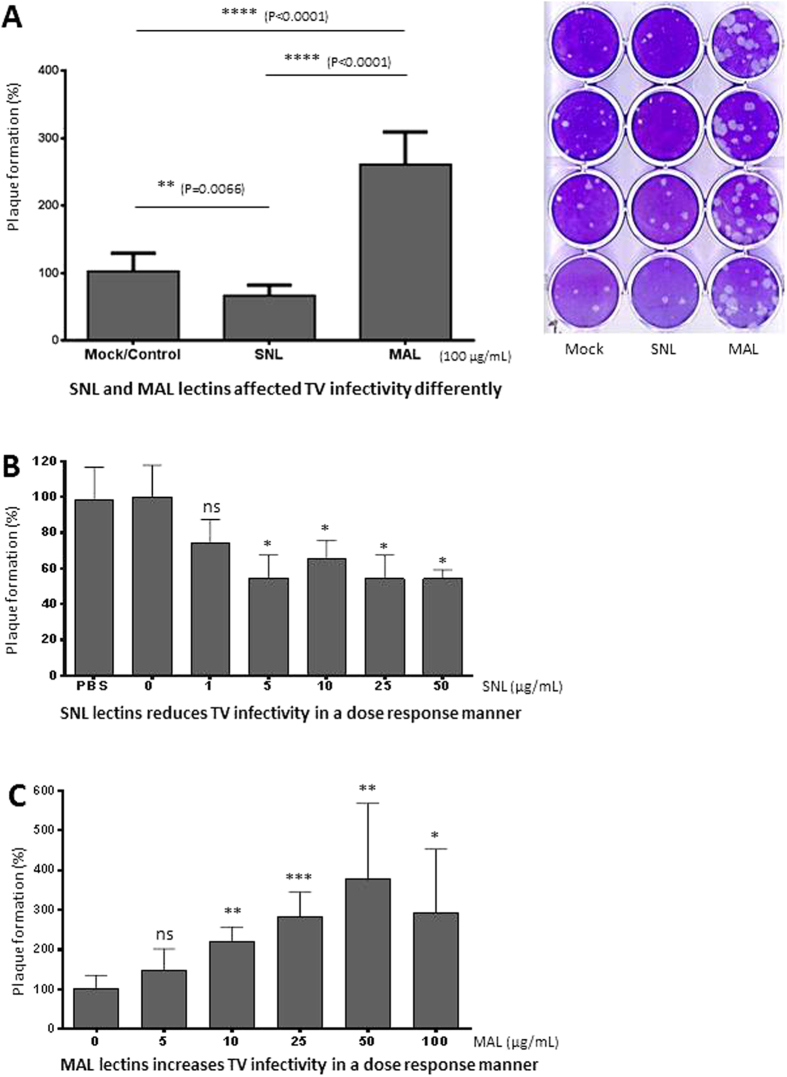
Sialic acid (SA)-binding lectins affected Tulane virus (TV) infectivity differently. (**A**) *Sambucus nigra* lectin (SNL) inhibited, while *Maackia amurensis* leukoagglutinin (MAL) increased TV infectivity in LLC-MK2 cells. Prior to TV infection, the LLC-MK2 cells were treated with SA-binding lectins; 100 μg/mL of SNL or MAL in 1x phosphate buffer saline (PBS, pH7.4). Mock control cells were treated with PBS only. The mean values (plaque numbers) of the mock/control group were set as 100%, while percentages (%) of the plaque numbers for individual wells of both mock/control and neuraminidase-treatment groups relative to the mean values of the mock/control group were calculated and shown. The statistical differences between data groups are indicated. Each experiment was repeated at least eight times. (**B** and **C**) Dose-response effects of SNL and MAL at different concentrations on TV infectivity in LLC-MK2 cells. The mean value (plaque numbers) of the control group without SNL (**B**) or MAL (**C**) treatment (0 μg/mL) was set 100%, while the reduction (**B**) or enhancement (**C**) rates (%) of the SNL/MAL treatment groups were calculated and shown. Each experiment was repeated at least four times. The statistical differences between the control groups (0 μg/mL SNL/MAL) and the SNL/MAL treatment groups are indicated. “ns”, “*”, “**”, and “***” indicate P values > 0.05, <0.05, <0.01, and <0.001, respectively.

The role of cell surface SAs on TV infectivity in LLC-MK2 cells were assessed following treatment with two commonly used SA-binding lectins, *Sambucus nigra* lectin (SNL) or *Maackia amurensis* leukoagglutinin (MAL). SNL is known to bind α-2,6 linked SAs, while MAL binds α-2,3 linked SAs on SGCs^50^. As expected, SNL at 100 μg/mL inhibited TV infection by ~32% (Fig. 3A, *P *= 0.0066), indicating that cell surface SGCs with α-2,6 linked SAs is important for TV infection. However, the low inhibition rate suggests that SGCs with SAs in other linkages also help TV infection. Surprisingly, MAL at the same concentration enhanced TV infection to 262% of the untreated control (P < 0.0001). Both SNL and MAL exhibited a dose-dependent response to TV infectivity (Fig. 3B,C). While SNL reached the plateau of inhibition effects at 5 μg/mL, MAL had the maximum enhancement effects at 50 μg/mL. The lectin-enhancements of viral infection have been observed previously, including mannose-binding lectin (MBL) on Ebola virus^51^ and galactose binding lectin-1 (Gal-1) on HIV^52^ (see Discussion).

### The MAL enhancement effects on TV infectivity were SA dependent

**Figure 4:**
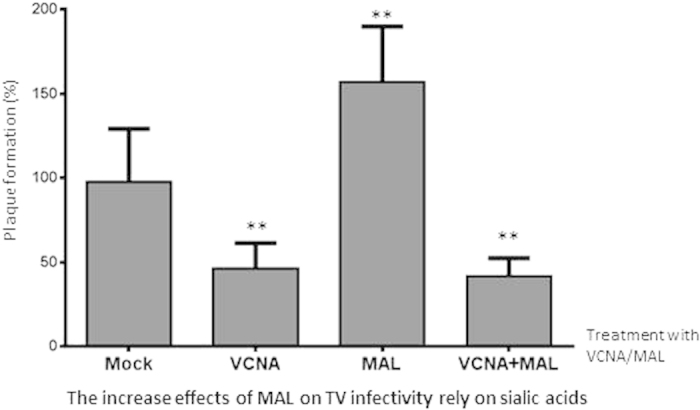
The enhancement effects of *Maackia amurensis* leukoagglutinin (MAL) on TV infectivity are abolished by neuraminidase treatment. LLC-MK2 cells were cultured in 12-well plates for plaque assays. Treatment of cells with *Vibrio cholerae* neuraminidase (VCNA, 60 mU/well) inhibits, while MAL treatment (100 μg/mL) of mixed TV/LLC-MK2 cells increases TV infectivity. The enhancement effects of MAL disappeared when the cells were pre-treated with VCNA. The mean values (plaque numbers) of the mock/control group were set as 100%, while percentages (%) of the plaque numbers for individual wells of mock/control, VCNA-, MAL-, and VANA+MAL-treated groups relative to the mean values of the mock/control group were calculated and shown. Each experiment was repeated at least four times. The statistical differences between the mock/control groups and the VCNA-, and VANA+MAL-treated groups are indicated. “**” indicates P values < 0.01.

Since MAL is a SA binding lectin, we examined whether the enhancement effects of MAL on TV infectivity are SA dependent. As expected, treatment of LLC-MK2 cells with neuraminidase from *Vibrio cholerae* (VCNA) stopped the enhancement effects of MAL on TV infectivity, leading to a net reduction in TV infection, similar to the reduction observed after treatment of neuraminidase alone (Fig. 4). These data indicate that the enhancement effects of MAL on TV infectivity rely on cell surface SAs.

### MAL treatment resulted in steady increases in TV replication

**Figure 5:**
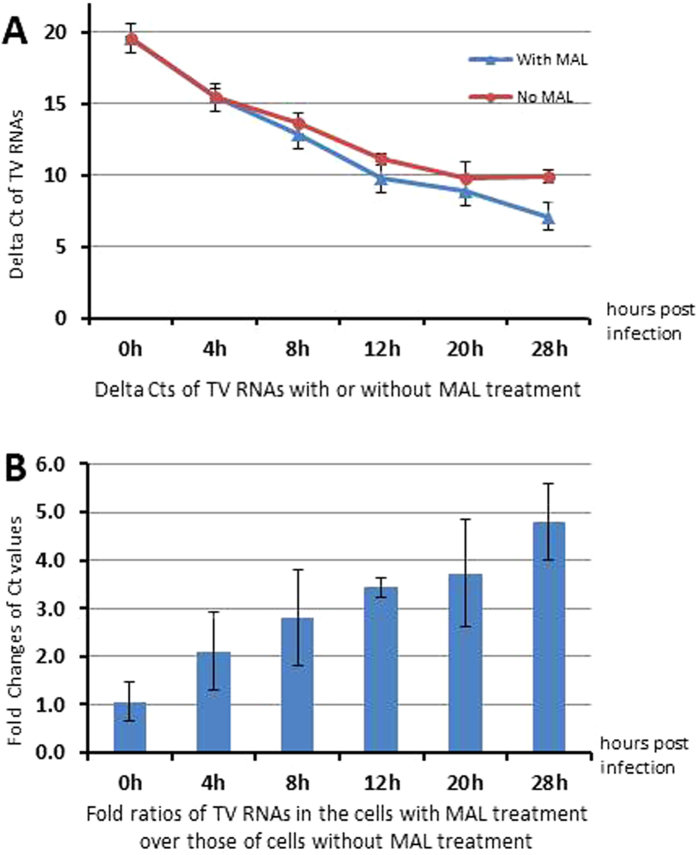
*Maackia amurensis* leukoagglutinin (MAL) steadily increased Tulane virus (TV) replication in LLC-MK2 cells during the first 28 hours post-infection. (**A**) Delta Ct dynamics of TV RNAs from infected cells with MAL (blue line) or without MAL (red line) treatment (100 μg/ml/well), as measured by quantitative RT- PCR (qRT-PCR). Delta Ct values are shown on the Y-axis, while the time points are indicated on the X-axis. (**B**) Fold ratios of TV RNAs from infected cells with MAL treatment compared to infected cells without MAL treatment. The Applied Biosystems® Eukaryotic 18S rRNA (4319413E) was used as an endogenous control to calculate the fold change by 2^DDCt. Each experiment was repeated four times.

To further understand the increased effects of MAL on TV infection and replication, we monitored TV replication over the first 28 hours after inoculation of LLC-MK2 cells with or without MAL treatment. Quantitative RT-PCR (qRT-PCR) showed increased TV RNA replication in MAL-treated compared with untreated cells at 4 hours post-inoculation (HPI) and the increase continued steadily for at least 28 HPI (Fig. 5A). At 28 HPI, TV RNA levels in MAL-treated cells were 4.8 fold higher than those of untreated cells (Fig. 5B). These data are consistent with the previous observation that MAL treatment enhanced TV infectivity. MAL may indirectly increase TV replication through the enhancement of virus binding/entry into cells.

### TV can infect SGC-positive but HBGA-negative LLC-MK2 cells

**Figure 6:**
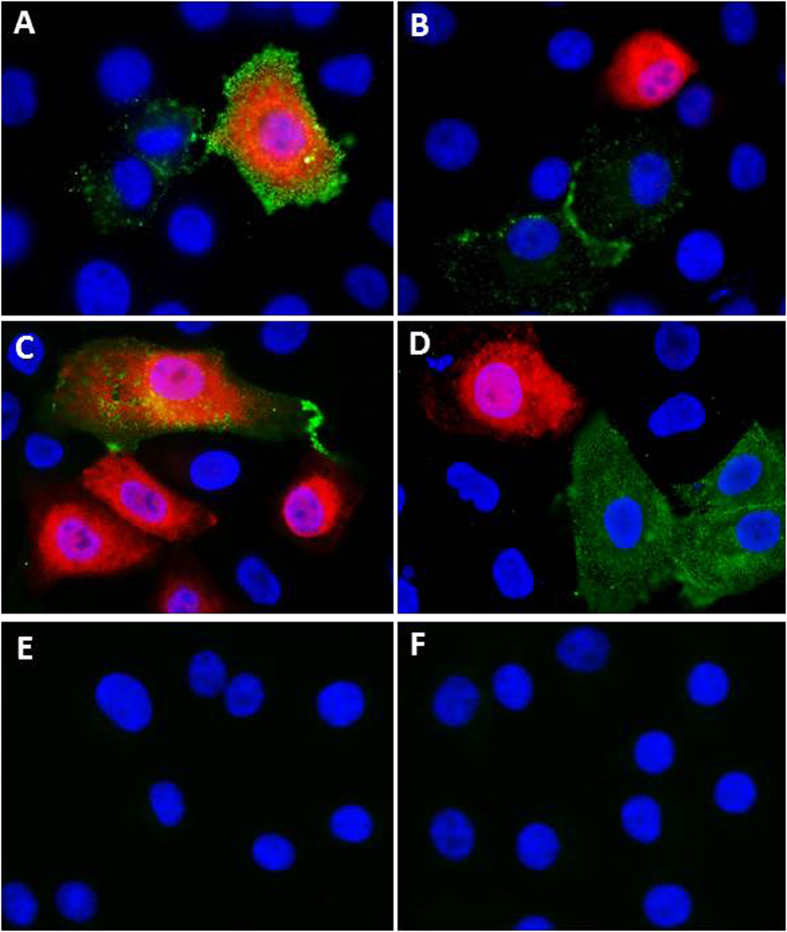
Histo-blood group antigen (HBGA) expression and Tulane virus (TV) infection of LLC-MK2 cells. The HBGA expression (green) of LLC-MK2 cells was determined through immunostaining using a panel of monoclonal antibodies (Mabs) against various HBGAs, which revealed only B antigen expression. Replicating TV virions (red) were detected by in-house hyperimmune rabbit serum against TV. Nuclei were stained with 4’,6-diamidino-2- phenylindole (DAPI, blue). (**A** to **D**) LLC-MK2 cells at 20 hours post-TV infection were stained using Mabs against B antigen (green) and hyperimmune serum against TV (red). Some B antigen-expressing cells were not infected with TV (**A**, **B** and **D**), while some TV-infected cells lacked detectable B antigen expression (**B**, **C** and **D**). (**E** and **F**) Cells were stained using the same procedure without the primary antibodies against B antigen and TV as negative controls.

**Figure 7:**
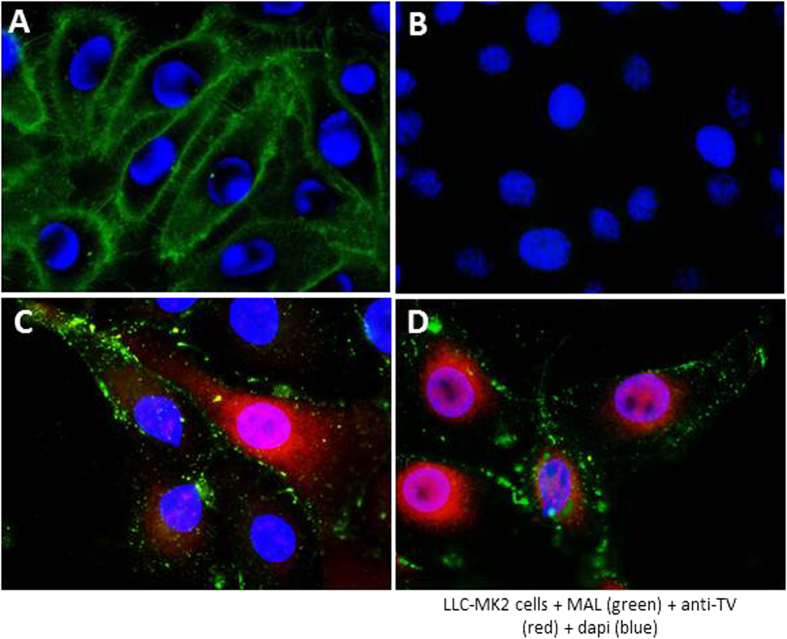
Expression of SGCs and Tulane virus (TV) infection of LLC-MK2 cells. (**A**) LLC-MK2 cells were stained with fluorescein-labeled *Maackia amurensis* leukoagglutinin (MAL, green). (**B**) Cells were stained via the same procedure as (**A**) without MAL (negative control). (**C** and **D**) LLC-MK2 cells at 15 hours post-TV infection were stained by fluorescein-labeled MAL (green) and hyperimmune rabbit serum against TV (red). MAL bound all cells and all TV-infected cells express SAs. Nuclei were stained with 4’,6-diamidino-2-phenylindole (DAPI, blue).

TV binds B and A type 3 antigens^13^,^53^. When the HBGA expression patterns of LLC-MK2 cells were examined using a panel of monoclonal antibodies (Mabs) specific to A, B, H, Le^a^, Le^b^, Le^x^, and Le^y^ antigens, only B antigen was detected in a small number (<15%) of cells at any given time point (Fig. 6, data not shown). On the other hand, the entire monolayer of LLC-MK2 cells was stained with fluorescein-labeled MAL or anti-GM1 antibody (Fig. 7A, data not shown), indicating SGC expression on all these cells. Co-localization of B antigens and TV by immunofluorescence microscopy indicated that TV infection was not always correlated with the expression of B antigen on the cells. For example, both TV-uninfected but with strong B antigen expression and TV-infected but without B antigen expression were seen (Fig. 6). On the other hand, all TV-infected cells appeared to be α,2-3-linked SA-positive (Fig. 7). Thus, TV can infect SGC-positive but HBGA-negative LLC-MK2 cells. It was noted that TV infection leads to striking difference in SGC distribution, resulting in clusters of SGCs on the TV-infected cells (Fig. 7, compared A with C and D).

### MAL interacted with TVs

**Figure 8:**
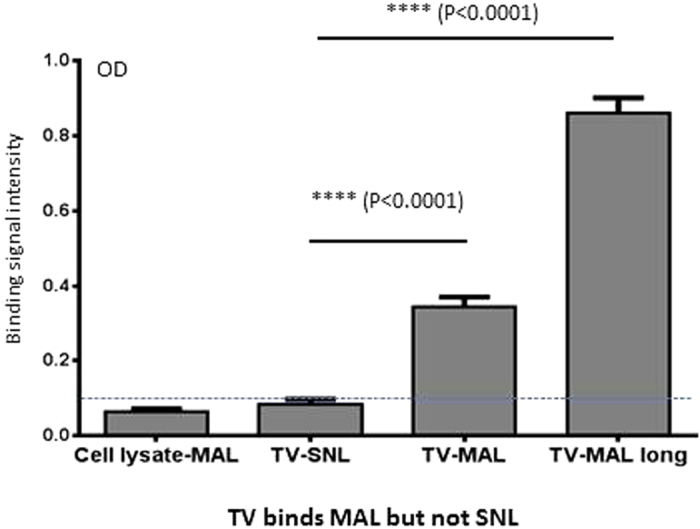
TVs interact with *Maackia amurensis* leukoagglutinin (MAL). CsCl gradient-purified TV (1 × 10^4^ PFU/mL) was coated on a microtiter plate and incubated with biotinylated MAL and *Sambucus nigra* lectin (SNL). Detection of bound biotinylated MAL/SNL by streptavidin-horse radish peroxidase (HRP) conjugates through ELISA showed interaction between TV and MAL (TV-MAL and TV-MAL long), but not SNL (TV-SNL). Coating the same gradient fraction of uninfected LLC-MK2 cell lysate and incubation with MAL revealed no signal (Cell lysate-MAL, OD <0.1) that serves as a negative control. Incubation of purified TV with MAL for longer time (overnight instead of one hour in the other experiments) revealed higher binding signals (TV-MAL long). A dashed line indicates the signal cutoff of 0.1. Each experiment was repeated at least four times. The statistical differences between data groups are indicated.

To further understand the mechanisms of the enhancement effects of MAL on TV replication, CsCl gradient-purified TV and corresponding fraction of cell lysate without TV was coated on plates and incubated with biotinylated MAL or SNL. Bound MAL, but not SNL was detected by streptavidin-HRP conjugates through ELISA (Fig. 8). Marginal binding signals (OD_450_ < 0.1) between the cell lysate fractions of uninfected LLC-MK2 cells and MAL were observed, which serve as a negative control. Thus, TV most likely interacts directly with MAL.

### Rapid internalization of MAL into LLC-MK2 and MA-104 cells

**Figure 9:**
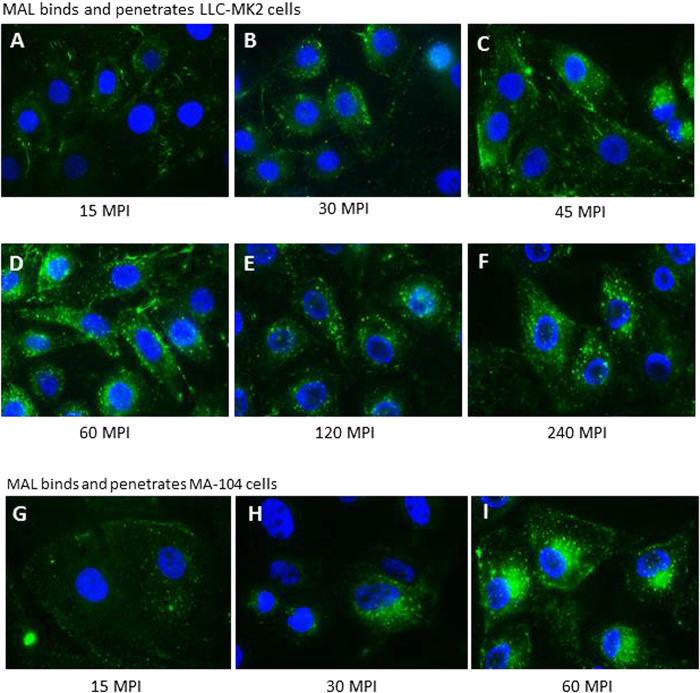
Dynamics of *Maackia amurensis* leukoagglutinin (MAL) bound to LLC-MK2 and MA-104 cells. Fluorescein-labeled MAL (green) was incubated with LLC-MK2 (A to F) or MA-104 (G to I) cells, and images were taken at different time points (15, 30, 45, 60, 120, and 240 minutes post-incubation, MPI) using fluorescence microscopy to observe the dynamic changes of the bound MAL. Nuclei were stained with 4’,6-diamidino-2-phenylindole (DAPI, blue). MAL was found to cluster in the cytoplasm of cells around 30 to 45 MPI.

Fluorescein-labeled MAL was incubated with cells of LLC-MK2 and MA-104 (another TV permissive cell line) separately and the dynamic changes of the cell-bound MAL were monitored at 15, 30, 45, 60, 120, and 240 minutes post-incubation (MPI). Shortly after binding to LLC-MK2 cells (15 MPI, Fig. 9A), the bound MAL started to internalize into the cells (Fig. 9, B to F). MAL tended to cluster in the cytoplasm around the nuclei. It remained detectable in cells for at least 18 hours (data not shown). This internalization may help the entry of TV that bound to MAL into LLC-MK2 cells (see discussion). Similar dynamics of MAL internalization were also observed after incubation of MAL with MA-104 cells (Fig. 9, G to I).

## Discussion

In this study, we provide the first evidence that TV recognizes SAs on cell surface SGCs based on multiple experiments with different approaches. In combination with the previously known TV-HBGA interactions^10^,^13^, TV appears similar to huNoVs that can also recognize SAs and HBGAs^15^,^38^,^54^. Since huNoVs still cannot be cultivated in cell culture, TV would serve as a valuable surrogate to study the roles of both SGCs and HBGAs and their coordination in the infection and pathogenesis of huNoVs in humans. Our data from this study, including direct binding between TV virions and SGCs by ELISA and the demonstration of an essential role for SGCs in TV infection and replication in cell culture, indicates that cell surface SGCs are host receptors or at least important host factors for TV infection.

Several caliciviruses have been reported to recognize SA-containing SGCs, such as feline calicivirus (FCV) from the *Vesivirus* genus^46^; porcine sapovirus (PSaV) from the *Sapovirus* genus^47^; and murine (MNV)^48^ and huNoVs^38^ from the *Norovirus* genus. These data suggest that interaction with SGCs could be a common feature of caliciviruses. However, the unique feature of TV and huNoVs that they bind SGCs in addition to HBGAs, suggesting a mechanism involving two carbohydrate host factors in the early stages of viral infection. Thus, further investigation to determine the potential coordinated roles of the two carbohydrate factors in the infection of TV and huNoVs (see below) is highly significant.

TV appears to recognize SGCs with SAs in specific structures and linkages. For example, TV bound Neu5Ac-PAA, but not Neu5Gc-PAA, while the binding of TV to 6’-sialyllacNAc, with an α-2,6 linked-SA, is significantly stronger than binding to 3’-sialyllactose, with an α-2,3-linked SA. These specific interactions were supported through the blockade of TV infection of LLC-MK2 cells by SNL, a lectin that recognizes an α-2,6-linked SA. Further investigation into the interactions between TV and SGCs with an α-2,8 linked-SA or an internal SA is ongoing, which will help further understand the specific interactions between TV and SGCs. In a recent study, two huNoVs (GII.4 and GI.3) were reported to interact with gangliosides^38^, including GM3, GM2, and GM1a/b. The strongest binding affinity was to GM3, which has the same saccharide sequences as the 3’-sialyllactose used in this study. However, one should keep in mind that there may be a distinction between TV/huNoV binding to synthetic SGCs and cell surface SGC molecules. Therefore, while TV and huNoV share the common feature of interaction with SGCs, their interacting specificities may be different and this will need to be clarified through future studies.

The fact that TV and huNoV recognize both HBGAs and SGCs as host factors raises the question on how these two types of carbohydrates coordinate each other during TV/huNoV infection. In this regard, the cultivable TV would provide a useful model to study this issue. Previous studies showed that the type B HBGAs in human saliva and synthetic type B oligosaccharide were able to block TV replication^10^,^13^, in which saliva had better blocking activity compared to synthetic oligosaccharides. In this study, we observed that neuraminidases and the SA-binding lectin (SNL) reduced TV infectivity, indicating that SGCs are also important for TV infection. However, it was noted that blocking or removal of either HBGAs or SGCs alone could not completely abolish TV infection, suggesting a possible complimentary effect for the two carbohydrates during TV infection. In fact, co-localization experiments of SGCs, HBGAs, and replicating TV in LLC-MK2 cells indicated that TV could infect LLC-MK2 cells without detectable B antigen, suggesting that the HBGA may not be absolutely required for TV infection in this cell line. This may also mean that the roles of HBGAs can be compensated for by SGCs at certain level during TV infection of LLC-MK2 cells.

However, the efficiency of TV infection through a single carbohydrate may be lower than that facilitated by both HBGAs and SGCs, because blocking either HBGAs or SGCs inhibited TV infectivity. Another possible explanation for the inhibition of TV infection by blocking either carbohydrate may be due to the close proximity of their two binding sites. As a result, blocking one binding site may also affect the function of the other binding site. This scenario may also explain the observation that type B saliva exhibited higher inhibitory effects compared to synthetic oligosaccharides containing the same type B antigen, because saliva is considered to have much larger molecules containing type B antigens than synthetic oligosaccharides.

Another major finding of this study is the enhancement of TV infection by MAL, an α-2,3 linked SA binding lectin derived from a tree species in the family *Fabaceae*. The enhancement effects of MAL apparently rely on cell surface SAs, because pretreatment of LLC-MK2 cells with neuraminidase wiped out the enhancement effects of MAL (Fig. 4). In fact, our data showed that MAL is able to specifically bind both LLC-MK2 cells (Fig. 7) and TV (Fig. 8). These special features allow MAL to recruit and enrich TV virions onto the host cell surfaces. After binding to host cells (LLC-MK2 and MA-104), MAL triggers internalization (Fig. 9) and thus brings TV into the host cell cytoplasm in a highly efficient manner. This explains the enhancement of TV infectivity by MAL treatment. In contrast, due to the lack of interaction with TV (Fig. 8), SNL binds and blocks the α-2,6 linked SA-containing cell surface SGCs and thus inhibits TV infectivity (Fig. 3). The enhancing effects of MAL in TV infectivity in the light of the dual binding of MAL to both cells and TV are unique. MAL is known to bind to cells in a high affinity via cell surface α-2,3 linked SAs. As a result, TV should bind to MAL via another domain that needs to be defined in the future.

Similar lectin-dependent enhancements of viral infectivity have been reported previously. For example, human mannose-binding lectin (MBL), a calcium-dependent (C-type) lectin that recognizes pathogen-specific surface glycans, was found to enhance Ebola virus (EBOV) infectivity under low complement conditions^51^. Mechanistic study demonstrated that MBL is able to bind both the N-linked glycan epitopes on EBOV surfaces and the transmembrane C-type lectin receptors on host cells^51^. Another example is the observation that galectin-1 promotes HIV-1 replication. Further study showed that the host-soluble galectin-1 is able to interact directly with glycans of the HIV envelope glycoprotein gp120 and CD4 on host cells^52^. In both cases, the lectins are able to recruit and enrich viruses to the surface of host cells, facilitating viral infection, a scenario similar to that for MAL observed in our study. However, unlike EBOV and HIV that are enveloped viruses with glycoproteins on the surface, TV is a nonenveloped virus that its capsid may or may not be glycosylated. Thus, how MAL interacts with TV remains to be studied in the future.

## Additional Information

**How to cite this article**: Tan, M. *et al.* Tulane virus recognizes sialic acids as cellular receptors. *Sci. Rep.* **5**, 11784; doi: 10.1038/srep11784 (2015).
